# Effects of Solution Treatment on the Microstructure and Mechanical Properties of UNS S32750/F53/1.4410 SDSS (Super Duplex Stainless Steel) Alloy

**DOI:** 10.3390/ma18235447

**Published:** 2025-12-03

**Authors:** Vasile Dănuț Cojocaru, Mariana Lucia Angelescu, Nicolae Șerban, Nicoleta Zărnescu-Ivan, Elisabeta Mirela Cojocaru

**Affiliations:** Faculty of Materials Science and Engineering, National University of Science and Technology Politehnica of Bucharest, 060042 Bucharest, Romania; dan.cojocaru@upb.ro (V.D.C.); nicolae.serban@upb.ro (N.Ș.); nicoleta.zarnescu@upb.ro (N.Z.-I.); elisabeta.cojocaru@upb.ro (E.M.C.)

**Keywords:** UNS S32750 super duplex stainless steel, solution treatment parameters, microstructure, mechanical properties

## Abstract

The influence of solution treatment time on the microstructural and mechanical properties of a super duplex stainless steel was investigated. A solution annealing treatment at 1120 °C was applied to the hot-rolled alloy, with soaking times varying between 10 and 30 min. The microstructural characteristics before and after solution treatment were examined using XRD and EBSD techniques by measuring lattice parameters and micro-strains, weight fraction, average grain size, and maximum misorientation angle. The experimental results showed that the constituent phases are δ-Fe and γ-Fe, regardless of the alloy state. The mechanical properties of the solution-treated alloy were evaluated by tensile testing, measuring the ultimate tensile strength (σ_UTS_), yield strength (σ_0.2_), fracture strain (ε_f_), and impact toughness (KCV). Increasing the solution treatment time from 10 min to 30 min leads to improved ductility and reduced mechanical strength, with the volume of the ferrite phase increasing, the average austenite grain size decreasing, and the maximum misorientation angle decreasing. This is due to the ability of ferrite to absorb stress and to the greater participation of grains in the deformation process. Important decreases in high elastic strains and residual stress fields after solution treatment were also noted.

## 1. Introduction

Super duplex stainless steels (SDSS) are known for their high mechanical strength and excellent corrosion resistance in various aggressive environments, including seawater, acids, and aqueous chlorides, being mainly recommended for the petrochemical, naval, and nuclear industries [1,2,3,4,5,6,7,8,9,10,11,12]. This combination of mechanical and anti-corrosion properties is due to the microstructure consisting of two phases, γ austenite (face-centered cubic lattice) and δ ferrite (body-centered cubic lattice), with an equivalent pitting resistance (PRE) higher than 40, where PRE = % Cr + 3.3% Mo + 16% N [13,14,15,16,17,18,19,20].

To obtain the best mechanical properties and good corrosion resistance, a phase balance of ~50% ferrite and 50% austenite is desirable. The metallurgy of super duplex stainless steel requires very strict control of composition and heat-treatment regimes during machining or welding [21,22,23,24,25,26,27].

Despite their excellent mechanical properties and corrosion resistance conferred by the high content of Cr (25–27%wt.), Ni (6–8%wt.), Mo (3–5%wt.), and N (0.2–0.35%wt.), precipitation of secondary phases during hot working or cooling of SDSS can cause deterioration of these specific attributes [28,29,30,31,32,33].

Among the secondary phases—sigma (σ) [34], chi (χ) [35,36], secondary austenite (γ_2_) [37,38,39], carbides (e.g., M_23_C_6_) [40,41,42], and nitrides (e.g., Cr_2_N) [43,44]—σ phase is the most deleterious, as the presence of very small amounts in the microstructure leads to strong embrittlement and a decrease in ductility, toughness, and corrosion resistance [45,46,47]. Sigma phase (σ) is a chromium/molybdenum-rich intermetallic phase that forms in stainless steel when the material is exposed to high temperatures (550–1000 °C) for a long time, usually during slow cooling. This intermetallic phase denudes the adjacent matrix of chromium, significantly reducing the impact toughness and corrosion resistance of the alloy [48]. The diffusion rates of Cr and Mo in ferrite are much faster than in austenite, making ferrite more unstable, so the sigma phase nucleates either directly from ferrite δ → σ at the δ/γ interface or at the δ/δ grain boundaries or through the eutectoid reaction δ → σ + γ_2_ in ferrite grains in association with γ_2_ [49,50].

It has been reported that, in duplex stainless steels, the σ phase can precipitate around 800–850 °C with the fastest precipitation rate, following a ‘C’-type kinetic curve. In high-alloy duplex steels, a higher content of alloying elements, such as Mo and W, shifts the temperature range of sigma phase precipitation towards higher temperatures [51,52].

Since the high content of alloying elements in SDSS extends the stability range to higher temperatures and causes complex transformations and precipitation phenomena, a higher solution annealing temperature (usually in the temperature range of 1080–1140 °C) and a sufficiently long time are required to dissolve any intermetallic compounds.

Extremely high temperatures should be avoided in order to limit the occurrence of excess ferrite in the final structure [53,54].

The best performance of forged SDSS is obtained in the solution-treated condition, usually carried out between 1080 and 1140 °C, with a holding time long enough to dissolve all precipitates and to assimilate the alloying elements into the solid solution and distribute them homogeneously. This results in an equilibrium composition in both ferrite (δ) and austenite (γ) [55]. Cooling from the solution annealing temperature must be fast enough to avoid precipitation of secondary phases, especially the sigma phase, with its embrittlement effect; therefore, solution annealing is followed by rapid quenching, most commonly in water, which restores the mechanical properties and corrosion resistance of the alloy.

SDSS alloys are mainly used in the form of flat products, and their manufacturing process generally consists of two repeated alternative stages: deformation by hot rolling and annealing/solution treatment [56]. During hot rolling, as a result of the different mechanical behavior of the two constituent phases (γ austenite and δ ferrite), non-uniform deformation may occur. This can cause stresses, localized strains, and micro-crack formation in ferrite, which spreads toward phase boundaries, leading to premature failure if the mechanical processing parameters (deformation temperature and deformation degree) are not properly selected. Both mechanical processing parameters are playing a crucial role as they strongly affect the occurrence of intermetallic phases such as carbides, nitrides, or sigma phase, which deteriorate corrosion resistance, ductility, toughness, strength properties, and overall performance of the material [57,58,59,60].

After mechanical processing, annealing/solution treatment at high temperatures is required to restore ductility, dissolve unwanted intermetallic phases, and re-establish a proper δ ferrite/γ austenite phase content balance. Annealing is also influenced by two parameters, namely temperature and time. These thermal processing parameters may also affect the occurrence of secondary phases, the proportion of γ austenite and δ ferrite phases, and alloying element distribution between the two phases, which in turn affects corrosion behavior [61].

These two stages (hot rolling and annealing/solution treatment) represent the thermo-mechanical processing route of SDSS alloy, and it is crucial they are studied together as they are interconnected and their parameters directly control the microstructure, content balance between γ austenite and δ ferrite phases, and defect formation in the material.

Therefore, finding a proper thermo-mechanical route is necessary to optimize manufacturing processing, avoid damaging phase formation, and ensure the mechanical integrity and corrosion resistance of the SDSS alloy.

The present work continues a recent one [16], related to UNS S32750 SDSS alloy deformability investigation, and aims to study the effect of annealing/solution treatment holding time on an intensely hot-deformed by rolling UNS S32750/EN 1.4410/F53 super duplex stainless steel (SDSS) alloy, establishing, in this way, the optimal thermo-mechanical processing parameters.

## 2. Materials and Methods

In this research work, a solution-treated super duplex stainless steel UNS S32750/F53 was studied. The experiments described are part of a larger research study, in which the influence of heat treatment temperature on the structure and properties of the F53 alloy was also studied.

In order to investigate the influence of solution treatment duration on the microstructural and mechanical properties of the alloy, the as-received (AR) alloy was processed by hot rolling (HR) at 1150 °C with a 70% total deformation degree (thickness reduction) by using a Mario di Maio LQR120AS rolling mill (Mario di Maio Inc., Milano, Italy). The mode and degree of plastic deformation were chosen depending on the parameters imposed by the beneficiary of the research project.

Then, the alloy was subjected to solution treatments at 1120 °C (ST1, ST2, and ST3) using different treatment durations between 10 and 30 min, with water quenching (WC), to avoid the occurrence of intermetallic precipitates.

Solution treatments were performed using a GERO SR 100 × 500 furnace (Carbolite-Gero, Neuhausen, Germany). The heating temperature for ST was constantly 1120 °C, and the holding time was varied between 10 and 30 min, as follows:

ST1: heating temperature 1120 °C and duration 10 min, water quenching.

ST2: heating temperature 1120 °C and duration 20 min, water quenching.

ST3: heating temperature 1120 °C and duration 30 min, water quenching.

**Figure 1:**
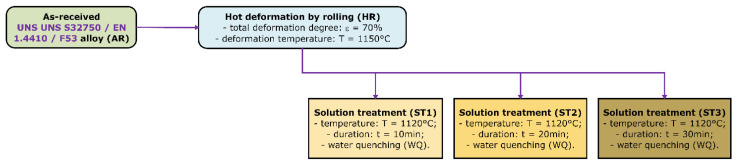
The thermo-mechanical processing routes applied to the UNS S32750/F53/1.4410 SDSS alloy.

Figure 1 shows the applied thermo-mechanical processing routes (TMP) for the UNS S32750/F53/1.4410 super duplex stainless steel alloy.

The evolution of microstructural parameters during solution treatment (ST) was studied by X-ray diffraction (XRD) and electron backscatter diffraction (EBSD) techniques by measuring the lattice parameters and lattice micro-strains (XRD), the weight fraction, the average grain size, and maximum misorientation angle (MO) in both ferrite and austenite phases.

The accumulated strain at the microstructural level was determined by the misorientation (MO) map distribution, which is based on the orientation deviation between a reference point and other points of the considered grain. As a reference point, it can be taken into account either the average orientation of the considered grain or the point of the grain where the KAM (kernel average misorientation) is the lowest [62,63,64].

The microstructure orientation (MO) map reveals grains with crystal orientations different from the average. This deviation, indicated by variations in color or shade on the map, can be caused either by accumulated deformation induced by grain sliding, twinning, or rotation or by other processes that may accompany plastic deformation, such as work hardening and dynamic recrystallization.

The X-ray diffraction analysis was carried out by using a RIGAKU MiniFlex600 (RIGAKU, Tokyo, Japan) benchtop diffractometer. The analysis technique involved evaluating the alloy’s X-ray diffraction patterns recorded between 2θ = 30° and 2θ = 100° using Cu-Kα radiation with a limit of detection of 0.1 to 1 wt. % for each phase.

Microstructural analysis was performed using a TESCAN VEGA II—XMU SEM microscope (TESCAN, Brno, Czech Republic). Samples were precisely cut with a Metkon MICRACUT 200 cutting equipment (Metkon Instruments Inc., Bursa, Turkey) using a diamond disk. All samples were hot mounted in conductive phenolic resin (NX-MET, Echirolles, France) and then polished with a MetkonDigiprep ACCURA machine (Metkon Instruments Inc., Bursa, Turkey). A Buehler VibroMet 2 vibratory polisher (Buehler, Lake Bluff, IL, USA) was used to apply a final, high-quality polishing step to improve the sample’s surface finish.

**Figure 2:**
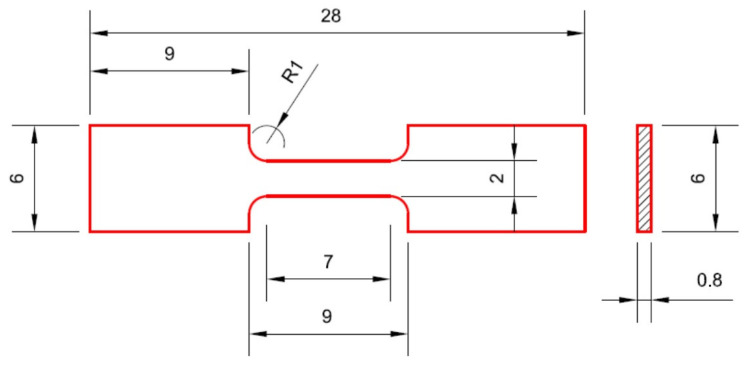
The geometric configuration of samples for mechanical testing [65].

The mechanical properties of the samples were analyzed using a GATAN MicroTest-2000N machine (Gatan Inc., Pleasanton, CA, USA). A strain rate of 1 × 10^−4^ s^−1^ was used. The specimens were “dog-bone” shaped. The calibrated part of the specimens had a width of 2 mm, a thickness of 0.8 mm, and a gauge length of 7 mm (Figure 2).

The mechanical properties measured from stress–strain curves are ultimate tensile strength (σ_UTS_); yield strength (σ_0.2_); elongation to fracture (ε); and absorbed energy (KCV), with all values rounded to the nearest integer.

## 3. Results and Discussion

### 3.1. Microstructural Analysis

#### 3.1.1. As-Received UNS S32750/EN 1.4410/F53 Super Duplex Stainless Steel (SDSS) Alloy

**Figure 3:**
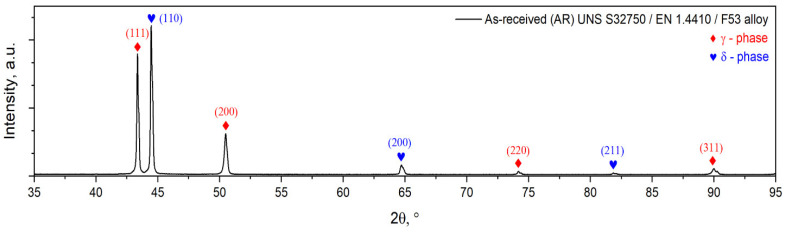
XRD spectra of as-received (AR) UNS S32750/F53/1.4410 SDSS alloy.

The X-ray diffraction (XRD) pattern of the as-received (AR) UNS S32750/EN 1.4410/F53 super duplex stainless steel (SDSS) alloy is illustrated in Figure 3. As observed, the XRD pattern shows the presence of both ferrite (δ-phase) and austenite (γ-phase) and the absence of any other secondary phase (i.e., σ, χ, etc.) in the material.

The ferrite (δ-phase) phase has a BCC (body-centered cubic) crystalline system, with a lattice parameter of a = 2.88(1) Å and a lattice micro-strain of ε = 0.03(6)%, while the austenite (γ-phase) phase has an FCC (face-centered cubic) crystalline system, with a lattice parameter of a = 3.61(5) Å and a lattice micro-strain of ε = 0.03(7)% (see Table 1).

**Figure 4:**
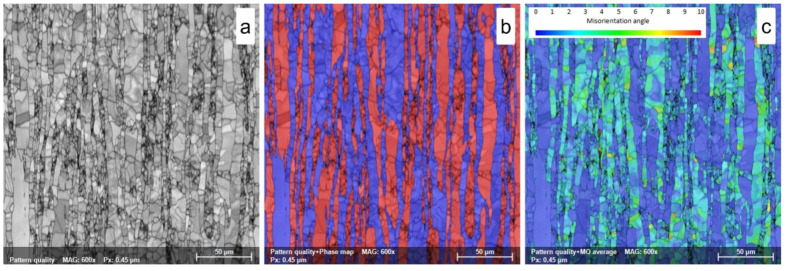
SEM-EBSD map of as-received (AR) UNS S32750/F53/1.4410 SDSS alloy; (**a**)—pattern quality (PQ) map; (**b**)—phase map; (**c**)—misorientation map.

The microstructure of the as-received (AR) UNS S32750/EN 1.4410/F53 SDSS alloy is shown in Figure 4a–c and is composed of a ferritic matrix colored in blue and an elongated island-like morphology austenite colored in red (see Figure 4a,b). The ferrite phase shows a weight fraction close to 55.1 ± 0.9%wt. and an average grain size close to D = 14.2 ± 1.3 μm, while the austenite phase has a weight fraction close to 44.9 ± 0.9%wt. and an average grain size close to D = 6.9 ± 1.9 μm (see Table 1).

Figure 4c illustrates the misorientation distribution map, which can be used as a qualitative instrument to assess the micro-strain. It shows that both ferrite and austenite phases exhibit a lower residual strain–stress field, characterized by a maximum MO (misorientation) angle close to 10° (9.1 ± 0.7)° in the ferrite case and (6.2 ± 0.5)° in the austenite case, respectively. However, when analyzing the misorientation distribution map, it can be observed that the residual strain–stress field is mostly located in the ferrite phase grains, which suggests that the ferrite phase is accumulating more internal stress than the austenite.

#### 3.1.2. Hot-Rolled UNS S32750/EN 1.4410/F53 Super Duplex Stainless Steel (SDSS) Alloy

**Figure 5:**
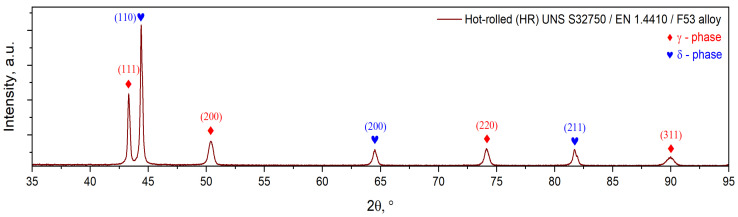
XRD spectra of hot-rolled (HR) UNS S32750/F53/1.4410 SDSS alloy.

The X-ray diffraction (XRD) pattern of the hot-rolled (HR) UNS S32750/EN 1.4410/F53 SDSS alloy is illustrated in Figure 5. As observed, the XRD pattern shows only the presence of ferrite (δ-phase) and austenite (γ-phase), with no other secondary phase.

The ferrite (δ-phase) phase was indexed in a BCC crystalline system, showing a lattice parameter close to a = 2.88(6) Å and a lattice micro-strain ε = 0.11(2)%, while the austenite (γ-phase) phase was indexed in an FCC crystalline system, showing a lattice parameter close to a = 3.61(9) Å and a lattice micro-strain ε = 0.34(2)%, respectively (see Table 2).

**Figure 6:**
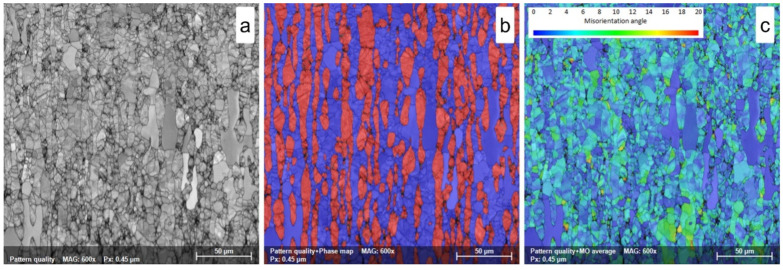
SEM-EBSD map of hot-rolled (HR) UNS S32750/F53/1.4410 SDSS alloy; (**a**)—pattern quality (PQ) map; (**b**)—phase map; (**c**)—misorientation map.

The microstructure of the hot-rolled (HR) UNS S32750/EN 1.4410/F53 SDSS alloy is shown in Figure 6a–c. As observed, the microstructure is composed of a mixture of ferrite and austenite, in which the matrix is constituted of the ferrite, while the austenite shows an island-like morphology, elongated along the rolling direction (RD) (see Figure 6a,b).

Also, one can observe that both ferrite and austenite grains show signs of intense deformation due to the high deformation degree (70% thickness reduction). However, in the case of ferrite, the presence of dynamically recrystallized (DRX) grains is obvious, indicating that the high deformation temperature favors the DRX mechanism. In comparison with the as-received (AR) state, the ferrite phase shows a decrease in weight fraction to a value close to 53.5 ± 0.8%wt., while the austenite phase shows an increase to a value close to 46.5 ± 0.8%wt. (see Table 2), as a consequence of the high deformation temperature (1150 °C).

The analysis shows that both ferrite and austenite phases have significant internal strain (Figure 6c), with a maximum MO of 19.1°± 1.6° in the ferrite and 12.3° ± 1.3° in the austenite, indicating a high residual strain–stress field. The MO map distribution (Figure 6c) displays large variations from the average grain orientation (KAM—kernel average misorientation) due to the accumulated strain from plastic deformation like slip, twinning, and rotation as well as effects like strain hardening and dynamic recrystallization.

Lower MO levels are recorded in austenite grains than in ferrite grains, revealing that, during intense plastic deformation, the austenite grains more easily accommodate the deformation due to the larger possibilities to activate slip/twinning in FCC crystalline structures (γ-phase) in comparison with the BCC crystalline structure of ferrite (δ-phase). However, due to the DRX phenomena, a lower residual strain field is registered in the recrystallized ferrite grains (max. MO is below 3°). No new dynamically recrystallized γ-phase grains are observed.

#### 3.1.3. Solution-Treated UNS S32750/EN 1.4410/F53 Super Duplex Stainless Steel (SDSS) Alloy

**Figure 7:**
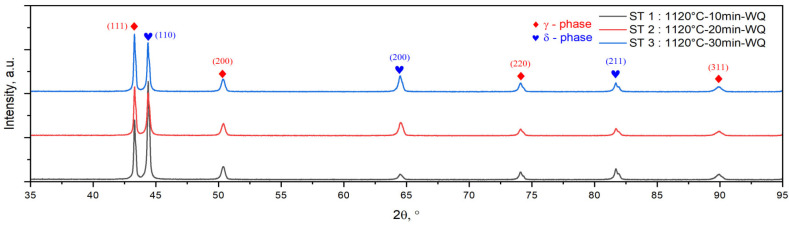
XRD spectra of solution-treated ST1 (1120 °C—10 min—WQ), ST2 (1120 °C—20 min—WQ), and ST3 (1120 °C—30 min—WQ) UNS S32750/F53/1.4410 SDSS alloy.

Figure 7 shows the X-ray diffraction (XRD) spectra of the solution-treated UNS S32750/F53/1.4410 SDSS alloy in all three states: ST1 (1120 °C—10 min—WQ), ST2 (1120 °C—20 min—WQ), and ST3 (1120 °C—30 min—WQ), respectively. As can be seen, the XRD pattern shows the presence of both ferrite (δ-phase) and austenite (γ-phase) in all states, ST1, ST2, and ST3, without any other secondary phase found in it, like in the case of both as-received (AR) and hot-rolled (HR) XRD spectra.

In all three solution treatment states, ST1, ST2, and ST3, the ferrite (δ-phase) has a body-centered cubic (BCC) crystalline system, with values of lattice parameter (a [Å]) and lattice micro-strain (ε [%]) of a = 2.88(1) Å and ε = 0.09(4)% for ST1 (1120 °C—10 min—WQ), a = 2.87(3) Å and ε = 0.08(1)% for ST2 (1120 °C—20 min—WQ), and a = 2.87(1) Å and ε = 0.08(2)% for ST3 (1120 °C—30 min—WQ), respectively. Regarding the austenite (γ-phase) phase, this has a face-centered cubic (FCC) crystalline system, with values of lattice parameter (a [Å]) and lattice micro-strain (ε [%]) of a = 3.61(4) Å and ε = 0.23(3)% for ST1 (1120 °C—10 min—WQ), a = 3.61(3) Å and ε = 0.23(8)% for ST2 (1120 °C—20 min—WQ), and a = 3.60(9) Å and ε = 0.23(8)% for ST3 (1120 °C—30 min—WQ), respectively (see Table 3).

Compared to the initial (AR) state, increasing the ST duration from 10 min to 30 min leads to an increase in the weight fraction of the ferrite phase up to a value close to 55.6 ± 0.5%wt., 57.4 ± 0.2%wt., and 58.5 ± 0.2%wt., respectively, while the weight fraction of the austenite phase shows a decrease to a value close to 44.4 ± 0.5%wt., 42.6 ± 0.2%wt., and 41.5 ± 0.2%wt., respectively (see Table 3).

**Figure 8:**
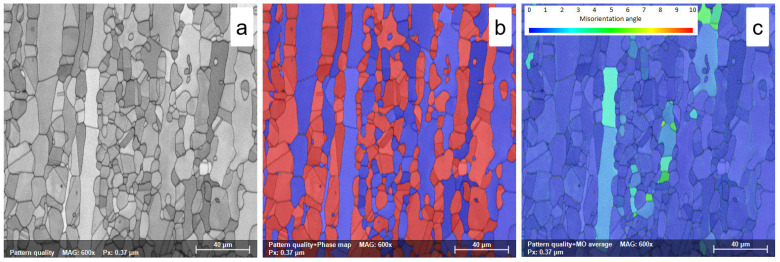
SEM-EBSD map of solution treated at 1120 °C—10 min—WQ (ST1) UNS S32750/F53/1.4410 SDSS alloy; (**a**)—pattern quality (PQ) map; (**b**)—phase map; (**c**)—misorientation map.

**Figure 9:**
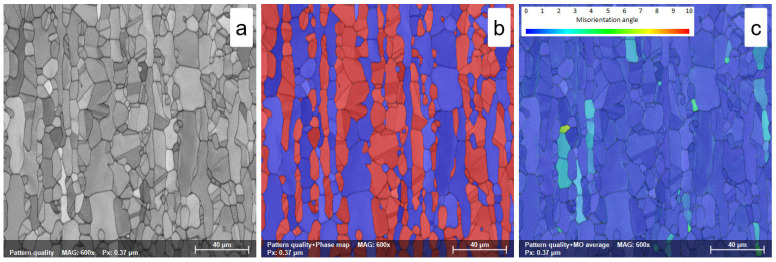
SEM-EBSD map of solution treated at 1120 °C—20 min—WQ (ST2) UNS S32750/F53/1.4410 SDSS alloy; (**a**)—pattern quality (PQ) map; (**b**)—phase map; (**c**)—misorientation map.

**Figure 10:**
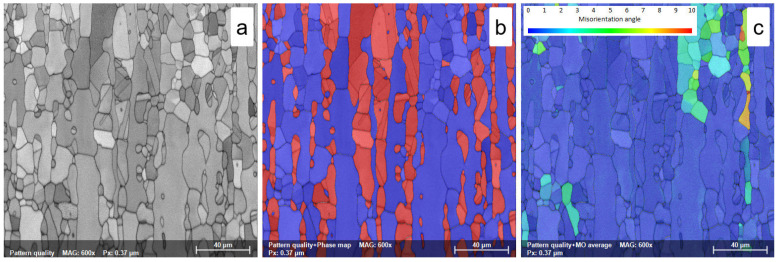
SEM-EBSD map of solution treated at 1120 °C—30 min—WQ (ST3) UNS S32750/F53/1.4410SDSS alloy; (**a**)—pattern quality (PQ) map; (**b**)—phase map; (**c**)—misorientation map.

In all solution treatment states, ST1 (1120 °C—10 min—WQ), ST2 (1120 °C—20 min—WQ), and ST3 (1120 °C—30 min—WQ), the morphology of both ferrite (δ-phase) and austenite (γ-phase) shows the typical characteristics of rolling structures, with austenite grains highly elongated along the RD direction within the ferrite matrix (Figure 8a,b, Figure 9a,b and Figure 10a,b).

In all cases, the microstructure shows that the solution treatment totally regenerates the previously intense deformed microstructure, leading to a microstructure free of all effects induced by the prior plastic deformation.

This effect is due to the high treatment temperature (1120 °C), which promotes the γ→δ phase transition. Increasing the solution treatment duration from 10 min to 20 min and, finally, to 30 min leads to a small increase in the average grain size of the ferrite phase, reaching a value close to 30.1 ± 0.6 μm, 31.1 ± 0.7 μm, and 32.2 ± 0.8 μm, respectively, while the average grain size of the austenite phase shows a small decrease due to the γ→δ phase transition, with values recorded close to 18.8 ± 1.1 μm, 18.5 ± 0.8 μm, and 18.1 ± 1.1 μm, respectively (see Table 3).

As observed in Figure 8c, Figure 9c and Figure 10c, after solution treatment, both ferrite and austenite phases have lower residual stress, with an average MO close to 3°. Increasing the solution treatment time from 10 to 30 min slightly decreases the maximum MO, while the austenite phase shows smaller maximum MO values than the ferrite phase (see Table 3). This stress reduction is attributed to the complete recrystallization of the previously deformed grains.

This observation suggests that an important effect induced by the solution treatments is the stress-relieving phenomena within the microstructure, which reduces internal residual stresses, preventing distortion and cracking and improving dimensional stability.

### 3.2. Mechanical Analysis

**Figure 11:**
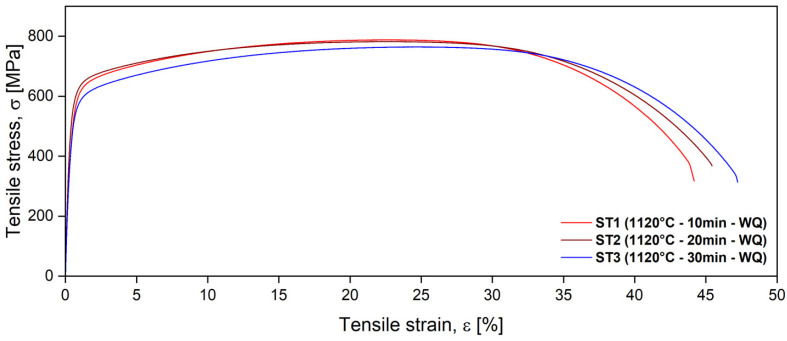
Typical strain–stress curves of solution-treated ST1 (1120 °C—10 min—WQ), ST2 (1120 °C—20 min—WQ), and ST3 (1120 °C—30 min—WQ) UNS S32750/F53/1.4410 SDSS alloy.

Tensile testing of the solution-treated alloy held at 1120 °C for 10, 20, and 30 min revealed that holding time affects its mechanical properties. The typical strain–stress curves of the solution-treated alloy are presented in Figure 11, and the results of the main mechanical properties, ultimate tensile strength (σ_UTS_), yield strength (σ_0.2_), fracture strain (ε_f_), and impact toughness (KCV), are summarized in Table 4.

As the holding time increased, the ultimate tensile strength decreased, while fracture strain increased. The decrease in strength is related to the metallurgical changes that occur during the prolonged holding time. With longer annealing times, the alloy’s microstructure can become more homogeneous, and grain growth may occur. These changes generally reduce the alloy’s ability to resist deformation and fracture. The good impact toughness of the solution-treated alloy is due to the absence of the sigma phase. The ST2 state provides a good combination of plasticity and strength properties. Toughness has not undergone any notable changes.

The influence of extending the solution annealing time is to increase ductility, which can also be explained by the increasing percent of ferrite, which is more capable of absorbing stress and preventing stress concentrations. Although austenite is more ductile than ferrite, a higher volume fraction of ferrite causes more grains to be involved in the plastic deformation process, which results in a more uniform deformation before fracture.

The mechanical properties are directly linked to the material’s microstructure. The analysis indicates that longer solution annealing times at 1120 °C lead to changes in the alloy’s microstructure, resulting in a material that becomes less strong and more ductile with longer holding times.

Analyzing the values of ultimate tensile strength and yield strength in Table 4, it can be seen that solution-treated UNS S32750/F53 super duplex stainless steel (SDSS) is a high-strength alloy with good plasticity, which offers the possibility of considerably reducing the thickness of the material, its formability being similar to that of austenitic stainless steel.

## 4. Conclusions

Solution treatment is a very important step in the processing of super duplex stainless steel UNS 32750/F53, improving its microstructural and mechanical properties. The evolution of microstructure and mechanical properties of a super duplex stainless steel UNS 32750/F53 showed that it was influenced during solution treatment at 1120 °C with a soaking time from 10 min to 30 min, as follows:

- The high soaking temperature and rapid water quenching prevented the formation of the harmful sigma phase, which can form during slow cooling at high temperatures.
- By increasing the duration of the solution treatment (at 1120 °C) from 10 min to 30 min, the following effects were recorded:
  - the average grain size of the ferrite phase increased;
  - the average grain size of the austenite phase slightly decreased;
  - the MO angle of both ferrite and austenite slightly decreased.
- Solution treatment stress-relieves the microstructure by lowering high internal elastic strains and residual stress fields, which are often introduced during processing like hot rolling or additive manufacturing. This process “re-generates” the microstructure by allowing atoms to rearrange. The reduction in residual stress is also a result of the material recovering and recrystallizing, which relieves stresses that build up during mechanical or thermal treatments.
- Increasing solution holding time from 10 min to 30 min improves ductility (ε_f_) while decreasing mechanical strength (σ_UTS_**)** due to microstructural changes. A longer holding time at the solution annealing temperature of 1120 °C allows for more complete diffusion of alloying elements and the formation of more ferrite.
- The effect of increasing the holding time at the solution annealing temperature of 1120 °C on the mechanical properties can be explained by the increasing percent of ferrite, which is more capable to absorb stress and to prevent stress concentrations, causing more grains to be involved in the plastic deformation process.
- The high yield strength of solution-treated UNS S32750/F53 super duplex stainless steel (≥550 MPa) and tensile strength (≥760 MPa) enable the use of thinner sections, while its good ductility (≥15–25% elongation) means it can be worked similarly to austenitic stainless steels.

The results obtained are useful for correctly establishing the heat treatment parameters of hot-deformed F53 alloy parts in order to obtain optimal microstructural and mechanical characteristics.

## Figures and Tables

**Table 1:** Microstructural parameters of as-received (AR) UNS S32750/F53/1.4410 SDSS alloy.

	Ferrite (δ-Phase)	Austenite (γ-Phase)
XRD measurements:		
- Lattice parameter, a [Å]	2.88(1)	3.61(5)
- Lattice micro-strain, ε [%]	0.03(6)	0.03(7)
EBSD measurements:		
- Weight fraction, [%wt.]	55.1 ± 0.9	44.9 ± 0.9
- Average grain size, D [μm]	14.2 ± 1.3	6.9 ± 1.9
- Max. misorientation, [°]	9.1 ± 0.7	6.2 ± 0.5

**Table 2:** Microstructural parameters of hot-rolled (HR) UNS S32750/F53/1.4410 SDSS alloy.

	Ferrite (δ-Phase)	Austenite (γ-Phase)
XRD measurements:		
- Lattice parameter, a [Å]	2.88(6)	3.61(9)
- Lattice micro-strain, ε [%]	0.11(2)	0.34(2)
EBSD measurements:		
- Weight fraction, [%wt.]	53.5 ± 0.8	46.5 ± 0.8
- Average grain size, D [μm]	-	-
- Max. misorientation, [°]	19.1 ± 1.6	12.3 ± 1.3

**Table 3:** Evolution of microstructural parameters during solution treatment (ST).

		Ferrite (δ-Phase)	Austenite (γ-Phase)
Solution treatment: 1120 °C—10 min—WQ (ST1)			
XRD measurements	Lattice parameter, a [Å]	2.88 (1)	3.61 (4)
	Lattice micro-strain, ε [%]	0.09 (4)	0.23 (3)
EBSD measurements	Weight fraction, [%wt.]	55.6 ± 0.5	44.4 ± 0.5
	Average grain size, D [μm]	30.1 ± 0.6	18.8 ± 1.1
	Max. misorientation, [°]	5.6 ± 0.7	3.4 ± 1.2
Solution treatment: 1120 °C—20 min—WQ (ST2)			
XRD measurements	Lattice parameter, a [Å]	2.87 (3)	3.61 (3)
	Lattice micro-strain, ε [%]	0.08 (1)	0.23 (8)
EBSD measurements	Weight fraction, [%wt.]	57.4 ± 0.2	42.6 ± 0.2
	Average grain size, D [μm]	31.1 ± 0.7	18.5 ± 0.8
	Max. misorientation, [°]	5.1 ± 0.3	3.3 ± 1.5
Solution treatment: 1120 °C—30 min—WQ (ST3)			
XRD measurements	Lattice parameter, a [Å]	2.87 (1)	3.60 (9)
	Lattice micro-strain, ε [%]	0.08 (2)	0.23 (8)
EBSD measurements	Weight fraction, [%wt.]	58.5 ± 0.2	41.5 ± 0.2
	Average grain size, D [μm]	32.2 ± 0.8	18.1 ± 1.1
	Max. misorientation, [°]	5.1 ± 0.8	3.3 ± 1.1

**Table 4:** Mechanical characteristics of the alloy, solution treated at 1120 °C with holding times of 10 min (ST1), 20 min (ST2), and 30 min (ST3), with water quenching.

	Ultimate Tensile Strength, σ_UTS_ [MPa]	Yield Strength, σ_0.2_ [MPa]	Elongation to Fracture, ε_f_ [%]	Absorbed Energy KCV [J]
Minimum	>(730–750)	>(530–550)	>25	>100
ST1 (1120 °C—10 min—WQ)	786.8 ± 5.2	560.6 ± 4.9	44.1 ± 1.9	132.5 ± 1.9
ST2 (1120 °C—20 min—WQ)	783.8 ± 2.1	573.7 ± 6.7	46.7 ± 1.4	133.9 ± 2.8
ST3 (1120 °C—30 min—WQ)	762.5 ± 5.5	572.8 ± 3.5	48.4 ± 2.4	133.2 ± 0.2

## Data Availability

The original contributions presented in this study are included in the article. Further inquiries can be directed to the corresponding author.
